# Androgen receptor regulates the proliferation of myoblasts under appropriate or excessive stretch through IGF-1 receptor mediated p38 and ERK1/2 pathways

**DOI:** 10.1186/s12986-021-00610-y

**Published:** 2021-09-15

**Authors:** Shaoting Fu, Xiaojing Lin, Lijun Yin, Xiaohui Wang

**Affiliations:** 1 Department of Exercise Physiology, School of Kinesiology, Shanghai University of Sport, 188 Hengren Road, Yangpu District, Shanghai, 200438 China; 2 Department of Kinesiology, College of Physical Education, Shanghai Normal University, Shanghai, 200234 China

**Keywords:** AR, Stretch, Myoblast proliferation, IGF-1R, p38, ERK1/2

## Abstract

**Background:**

Androgen receptor (AR) exerts important roles in exercise-induced alterations of muscle mass, in which the proliferation and differentiation of satellite cells or myoblasts are crucial. Our previous study in C2C12 myoblasts demonstrated that 15% (mimic appropriate exercise) and 20% (mimic excessive exercise) stretches promoted and inhibited the proliferation respectively; and AR played a crucial role in 15% stretch-induced pro-proliferation through IGF-1-modulated PI3K/Akt, p38 and ERK1/2 pathways, but AR’s role in stretches-modulated proliferation of general myoblasts, especially 20% stretch, remains unclear, and the mechanisms need to be further clarified.

**Methods:**

Firstly, the discrepancy in proliferation and the above indicators between L6 (without AR) and C2C12 (with AR) myoblasts were compared under 15% or 20% stretch. Then the influences of transfection AR or exogenous IGF-1 treatment on proliferation and these indicators were detected in stretched L6 myoblasts.

**Results:**

(1) Under un-stretched state, the proliferation of L6 was slower than C2C12 cells. Furthermore, AR knockdown in C2C12 myoblasts repressed, while AR overexpression in L6 myoblasts promoted the proliferation. (2) 15% stretch-induced increases in the proliferation and activities of p38 and ERK1/2 were lower in L6 than C2C12 cells; AR overexpression enhanced the proliferation of 15% stretched L6 cells accompanied with the increases of p38 and ERK1/2 activities. (3) 20% stretch-induced anti-proliferation and inhibition of p38 activity were severer in L6 than C2C12 myoblasts; AR overexpression reversed the anti-proliferation of 20% stretch and enhanced p38 activity in L6 myoblasts. (4) In stretched L6 myoblasts, AR overexpression increased IGF-1R level despite no detectable IGF-1; and recombinant IGF-1 increased the proliferation, the level of IGF-1R, and the activities of p38 and ERK1/2 in 15% stretched L6 myoblasts.

**Conclusions:**

The study demonstrated AR's crucial roles in stretches-regulated proliferation of myoblasts, and increased AR fulfilled 15% stretch's pro-proliferation via activating IGF-1R- p38 and ERK1/2 pathways while decreased AR achieved 20% stretch's anti-proliferation via inhibiting IGF-1R- p38 pathway, which is useful to understand in depth the role and mechanisms of AR in appropriate exercise increasing while excessive exercise decreasing muscle mass.

## Introduction

Testosterone is widely reported to affect muscle mass, with high level testosterone promoting whereas low level declining muscle mass, mainly via the mediation of androgen receptor (AR)[1, 2]. Decrements in serum testosterone or muscle AR during aging are associated with muscle loss[3], and general or muscle-specific AR deletion leads to the decline in muscle mass and strength[4–6]. Selective androgen receptor modulators (SARMs) rather than large dose of androgens are employed to increase muscle mass and strength, as androgen-induced severe adverse effects on cardiovascular system, prostate hyperplasia and so on[7–9]. In fact, not only in sedentary states, but also in acute or chronic exercise conditions, elevated AR content in skeletal muscle is closely associated with exercise-induced muscle hypertrophy and strength gain [10], and AR blockade or myofiber-specific AR deletion attenuates exercise-induced increases in muscle mass and strength[11, 12]. Our previous work also indicated that in both resistance training and endurance training, AR plays a crucial role in training-induced muscle hypertrophy of rats using AR specific antagonist flutamide[13]. In addition, overtraining reduces the level of AR, which may be related to overtraining-induced decline in muscle mass [14].

In skeletal muscle, AR is predominantly expressed in satellite cells (skeletal muscle-derived stem cells) and myonuclei, therefore satellite cells are considered as the direct target of androgens. Androgen/AR's roles in promoting the proliferation of satellite cells or myoblasts have been well demonstrated in sedentary state in vivo[15] and under un-stretched condition in vitro [16]. Our previous study indicated that in C2C12 myoblasts subjected to 15% and 20% cyclic mechanical stretches at 0.5 Hz frequency lasting for 6 h, the levels of AR were increased and decreased respectively, accompanied with pro-proliferation and anti-proliferation respectively, and AR antagonist flutamide reversed the pro-proliferation of 15% stretch on C2C12 myoblasts[17], indicating the important effects of AR on stretches-modulated myoblast proliferation. But AR's roles in exercise-modulated muscle mass or myoblast proliferation need to be demonstrated and the molecular mechanisms require to be clarified. Totally different from C2C12 myoblasts which express AR, rat-derived L6 myoblasts have no detectable AR at transcription and translation levels[18]. So the discrepancy between L6 and C2C12 cells in proliferation under different stretches, and overexpression AR in L6 myoblasts as well as inhibition of AR and its downstream molecules could provide evidences for AR's role and its mechanisms.

Insulin-like growth factor (IGF-1) is an important regulator in muscle mass and strength through promoting the proliferation of satellite cells or myoblasts, increasing protein synthesis and decreasing protein degradation. It has been demonstrated that exogenous IGF-1 could significantly induce the proliferation of myoblasts in vivo and in vitro in a dose-dependent manner[19]. IGF-1 acts via binding to IGF-1 receptor (IGF-1R), and the proliferation of myoblasts is inhibited in muscle-specific IGF-1R knockout mice[20]. Upon IGF-1 binding to IGF-1R, two major signal pathways are activated: phosphoinositide 3-kinases (PI3K)/Akt (also known as protein kinase B) and mitogen-activated protein kinase (MAPK) pathways including p38 and extracellular signal-regulated kinases 1 and 2 (ERK1/2)[17]. Studies in primary satellite cells or myoblast cell lines demonstrated that the stimulatory effect of IGF-1 on proliferation was mediated by PI3K/Akt and MAPKs (p38 and ERK1/2) pathways, for specific inhibitors of PI3K (LY294002), Akt (KP372-1) and ERK1/2 (PD98059) attenuated the pro-proliferation of IGF-1, respectively [19, 21]. In fact, not only in un-stretched myoblasts, our previous work also found that in 15% mechanical stretched C2C12 myoblasts, the pro-proliferative effect of IGF-1 was realized via activating PI3K/Akt as well as p38 and ERK1/2 signal pathways [17]. However, whether the IGF-1/IGF-1R mediated PI3K/Akt, p38 and ERK1/2 signal pathways were involved in 20% stretch-induced anti-proliferation remains unclear.

For the relationship between AR and IGF-1/IGF-1R, numerous evidence indicate the crosstalk between androgen/AR and IGF-1/IGF-1R. Firstly, IGF-1/IGF-1R are the target genes of AR, and AR's effects in modulating myoblast proliferation and muscle hypertrophy are mainly mediated by IGF-1/IGF-1R, because androgen response elements (AREs) exist in IGF-1 promoter and androgen/AR agonist-induced enhancements in satellite cell activation and muscle regeneration are associated with the increase of IGF-1[22]; in addition, testosterone's effect on levator ani muscle hypertrophy and androgen/AR's role in promoting the proliferation of human skeletal muscle cells are both blocked by inhibiting IGF-1R[23]. Secondly, in turn, AR's level and activity could be elevated by IGF-1 in time- and dose-dependent manners in differentiating C2C12 cells[24, 25].

Therefore, the present study firstly compared the discrepancy between L6 and C2C12 myoblasts subjected to 15% and 20% stretches in proliferation, levels of IGF-1 and IGF-1R, and activities of PI3K/Akt, p38 and ERK1/2, then measured the above indicators in L6 myoblasts again after transfection with AR overexpression plasmid or treatment with IGF-1 recombinant polypeptide, for demonstrating the roles of AR in stretches-regulated proliferation of myoblasts and further exploring the possible mechanisms: IGF-1 mediated alterations in the activations of PI3K/Akt and MAPKs (p38 and ERK1/2)?

## Material and methods

### Cell culture

Rat-derived L6 myoblasts and mouse-derived C2C12 myoblasts were purchased from Chinese Academy of Sciences Cell Bank, and cultured in high-glucose Dulbecco’s modified Eagle medium (DMEM) (Gibco, USA) supplemented with 10% fetal bovine serum (FBS) (Gibco, USA) as well as 100 U/ml penicillin and 100 μg/ml streptomycin (Gibco, USA) at 5% CO2 and 37 ℃. All cells used in the experiments were at low passages (from passage 3 to passage 8).

### Plasmid construction and cell transfection

Full-length AR cDNA was amplified by PCR using specific primers (Forward: 5′-CTTGGTACCGAGCTCGGATCCGCCACCATGGAGGTGCAGTTAGGGC-3′ and Reverse: 5′-TGCTGGATATCTGCAGAATTCTCCACTGTGTGTGGAAATAGATGGG-3′) and inserted into pcDNA3.1 ( +) vector (Invitrogen) using restriction sites *EcoRI* and *BamHI* to generate pcDNA3.1-AR recombinant plasmid. All constructs used for experiments were sequenced.

For AR overexpression, L6 cells were seeded onto 6-well plates, and pcDNA3.1-AR recombinant plasmid (2.5 μg/well) were transfected using LipoPlus™ Reagent (Sage creation science, Beijing, China) according to the manufacturer's instructions. The equivalent amount of pcDNA3.1 vector was transfected as control. To confirm the efficiency of transfection, the level of AR was determined by Western blot at 24 h after transfection finished.

### RNA interference

For knockdown the level of AR in C2C12 cells, siRNA targeting AR (siRNA-AR) (sc-29203) and a scrambled negative control (siRNA-ctrl) (sc-37007) were purchased from Santa Cruz Biotechnology, and transfected into C2C12 cells using Lipofectamine 2000 reagent (Invitrogen, Carlsbad, CA, USA) according to manufacturer's protocol. The cells were incubated with siRNA mixture for a period of 36 h before being subjected to other assays.

### Application of cyclic mechanical stretches

C2C12 and L6 myoblasts were stretched as described in our previous studies[17, 26]. Briefly, cells were plated onto flexible-bottomed 6-well plates pre-coated with type-I collagen (BioFlex, FlexCell International Corporation, USA) at a density of 1 × 10^5^/well density and incubated for 24 h before exposing to mechanical strain. Cells were then subjected to cyclic mechanical stretch of 15% or 20% elongation at 0.5 Hz frequency for 6 h using a computer-controlled vacuum stretch apparatus (FX-5000 T Tension System, FlexCell International Corporation). In parallel with identical experimental conditions, cells grown in flexible-bottomed 6-well plates but left un-stretched were considered as control.

### Detection of cell proliferation

Cell proliferation was measured by Cell Counting Kit-8 (CCK8) (Dojindo Laboratories, Kumamoto, Japan) following the manufacturer’s protocols. At 24 h after mechanical stretches or transfection finished, culture medium was replaced with fresh cell culture medium, appropriate CCK8 was added into the medium in proportion, then the optical density (OD) values were detected at 450 nm after incubation for 2.5 h using a microplate reader (Biotek, Winooski, VT, USA).

### Determination of IGF-1 concentration

Once stretch finished, culture medium was replaced by DMEM without FBS immediately and incubated for 24 h, then cellular supernatant was collected and centrifuged at 12,000 rpm for 5 min at 4 ℃. IGF-1 concentration in cellular supernatant was determined by mouse/rat IGF-1 Quantikine ELISA Kit (R&D Systems Inc, Minneapolis, MN, USA) according to manufacturer’s instructions.

### Treatment with IGF-1 recombinant polypeptide

To confirm the role of IGF-1 in the proliferation of stretched myoblasts, and its relationship with PI3K/Akt and MAPKs (p38 and ERK1/2), IGF-1 recombinant polypeptide with different concentrations (200, 500, 1000 ng/ml)[17, 26] were added into culture medium before 15% stretch.

### Western blot

Cells after culture and treatments were collected and lysed in RIPA lysis buffer (Beyotime Biotechnology, Shanghai, China) containing protease and phosphatase inhibitor cocktail (Beyotime Biotechnology, Shanghai, China) and incubated on ice for 30 min, then centrifugated at 4 ℃, 12000 rpm for 20 min. The protein concentration in the supernatant was determined using BCA protein assay kit (Beyotime Biotechnology, Shanghai, China). ~30 μg protein were separated on SDS-PAGE gel, and subsequently transferred to the PVDF membranes (Millipore, Darmstadt, Germany). After blocking with 5% (w/v) fat-free milk at RT (~25 ℃) for 2 h, the membranes were incubated with primary antibodies at 4 ℃ overnight: AR (sc-816, 1:500), IGF-1R (AF-305, 1:500), p-PI3K [p85 (Tyr 458)/p55 (Tyr 199), 4228S, 1:1000], PI3K (4257P, 1:1000), p-Akt (Ser 473, 4060S, 1:1000), Akt (4691P, 1:1000), p-p38 MAPK (Thr 180/Tyr 182, 4511S, 1:1000), p38 (8690P, 1:1000), p-p44/42 MAPK (Thr 202/Tyr 204, 4370P,1:1000), p44/42 MAPK (4695P, 1:1000). Thereafter, the membranes were incubated with HRP-conjugated anti-mouse/rabbit /goat secondary antibodies at RT for 2 h, and the blots were visualized by ECL reagent (Merck Millipore, America) and detected by automatic chemiluminesence image analysis system (Tannon 5200, Tannon Technology Co., Ltd, China).

### Statistical analysis

All the experiments were repeated at least three times. Data were presented as mean ± standard deviation (SD), and statistical analysis was performed by SPSS 21.0. Statistical differences among experimental groups were determined by two-way ANOVA, and differences between two groups were analyzed by using Student’s t test. *p* < 0.05 was consider as statistically significant.

## Results

### Under un-stretched state, AR deficiency slowed down the proliferation rate of myoblasts

**Fig. 1:**
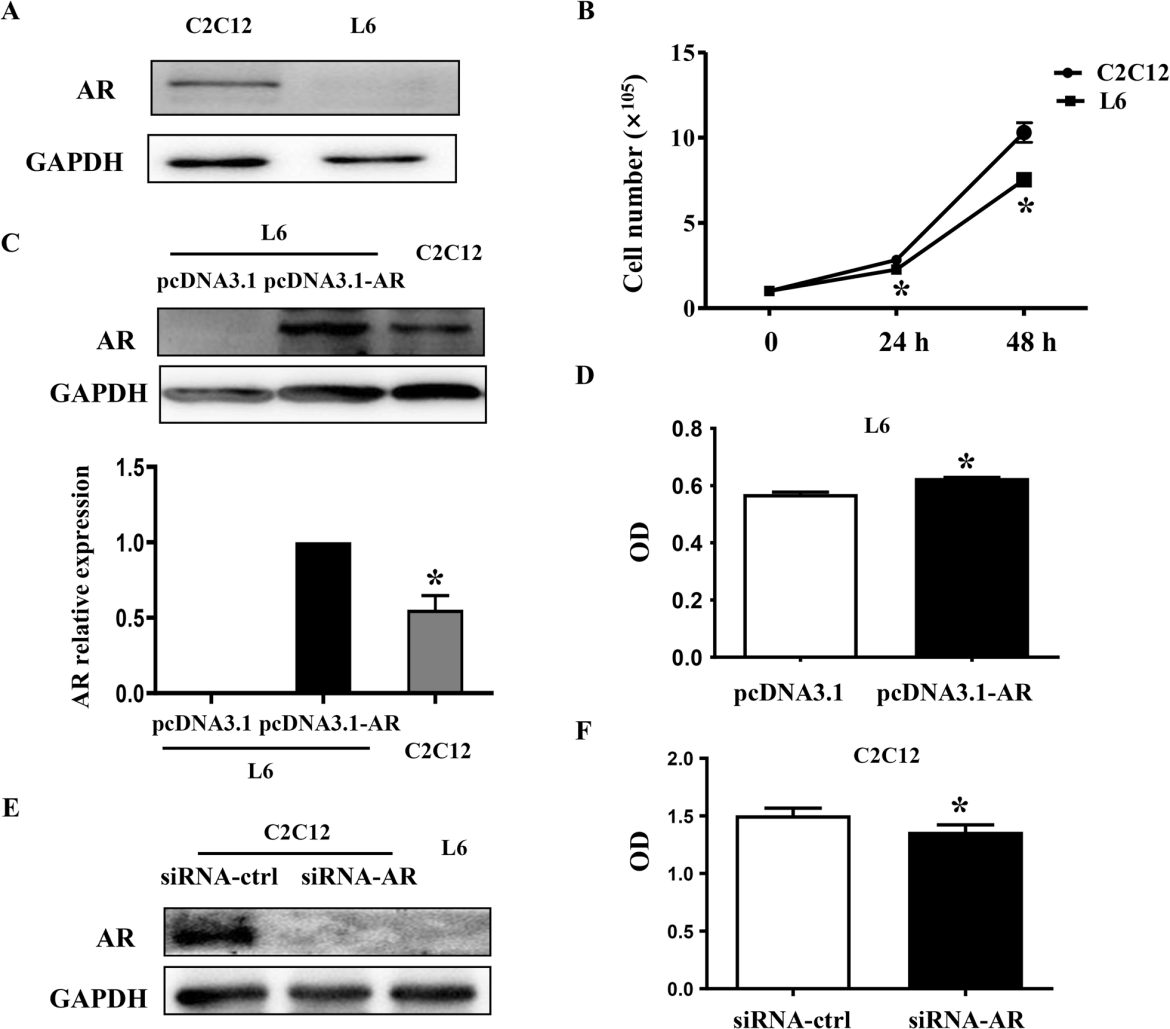
Under un-stretched state, AR deficiency slowed down the proliferation of myoblasts. **a** The difference in the level of AR and **b** cell number between C2C12 and L6 myoblasts were determined by western blot and cell counting, respectively. *p < 0.05 vs C2C12 myoblasts at the corresponding time point. **c** AR overexpression plasmid (2.5 ug/well) was transfected into L6 myoblasts, and the level of AR protein was determined and compared with C2C12 cells, and **d** cell proliferation was detected by CCK8. *p < 0.05, the proliferation of L6 myoblasts transfected with pcDNA3.1-AR vs pcDNA3.1 vector; **e** siRNA-AR (100 pmol/well) was transfected into C2C12 myoblasts using Lipofectamine 2000 reagent, and the level of AR protein was determined and compared with L6 myoblasts, and **f** cell proliferation was detected. *p < 0.05, the proliferation of C2C12 myoblasts transfection with siRNA-AR vs siRNA-ctrl. The experiments were repeated at least three times, and the results were from three independent experiments and represented as mean ± SD

We firstly confirmed that AR expressed in C2C12 myoblasts whereas no detectable AR was examined in L6 myoblasts (Fig. 1a), consistent with previous studies. Then the proliferation of C2C12 and L6 cells under un-stretched state were compared, and observed that the proliferation rate of L6 cells was significantly lower than that in C2C12 cells, as evidenced by the number of L6 myoblasts were about 80.6% and 72.5% of C2C12 myoblasts at 24 h and 48 h after seeding, respectively (Fig. 1b). To further clarify AR’s roles in myoblasts proliferation, we explored the influences of AR transfection into L6 myoblasts and knockdown of AR in C2C12 myoblasts on their proliferation, and found that the proliferation of L6 myoblasts was promoted (Fig. 1d) by transfection with AR (the level of AR was higher than that of C2C12 myoblasts) (Fig. 1c), while the proliferation of C2C12 myoblasts was inhibited (Fig. 1f) by AR knockdown utilizing siRNA AR (almost no detectable AR, similar to L6 cells) (Fig. 1e). These results demonstrated that AR plays an important role in myoblast proliferation under un-stretched state.

### The degrees of 15% stretch-induced pro-proliferation and 20% stretch-induced anti-proliferation in L6 myoblasts were different from that in C2C12 myoblasts

**Fig. 2:**
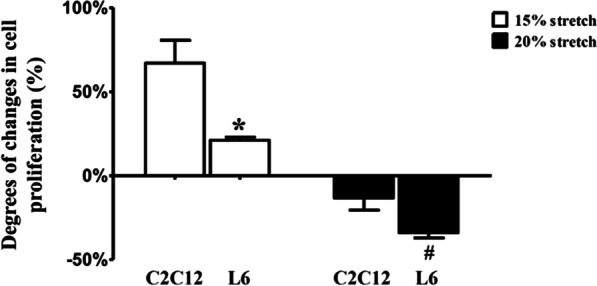
The degrees of pro- and anti-proliferation were different between L6 and C2C12 myoblasts undertaken 15% and 20% stretches. L6 and C2C12 myoblasts were seeded onto flexible-bottomed 6-well plates and divided into CON, 15% stretch, and 20% stretch groups. After 24 h incubation, they were subjected to 15% or 20% stretch at 0.5 Hz duration for 6 h. Then the cell proliferation was determined by CCK8 at 24 h after stretch finished, so the duration of the entire experiment was ~ 54 h. The alteration degrees of 15% stretch-induced pro-proliferation and 20% stretch-induced anti-proliferation were compared between C2C12 and L6 myoblasts using the following formula: = (OD_15% or 20% stretch_—OD_CON_)/OD_CON_. The experiments were repeated at least three times (mean ± SD, n = 3), * and ^#^ indicated *p* < 0.05 vs corresponding C2C12 cells

Our previous work has reported that 15% and 20% stretches promoted and inhibited the proliferation of C2C12 myoblasts (with AR expression), respectively [17]. In the current study, the proliferation of L6 myoblasts (without AR expression) undertaken 15% or 20% stretch was detected, and we were surprised to found that 15% and 20% stretches promoted and inhibited (rather than no influence) the proliferation of L6 myoblasts, respectively; but compared to C2C12 myoblasts, 15% stretch-induced pro-proliferative effect on L6 myoblasts was much lower (less than half of C2C12 cells), while 20% stretch-induced anti-proliferation on L6 myoblasts was obvious higher (above twofold of C2C12 cells) (Fig. 2), which indicated an important role of AR in stretch-modulated proliferation of myoblasts.

### Overexpression AR promoted 15% stretch-induced pro-proliferation, and reversed 20% stretch-induced anti-proliferation in L6 myoblasts

**Fig. 3:**
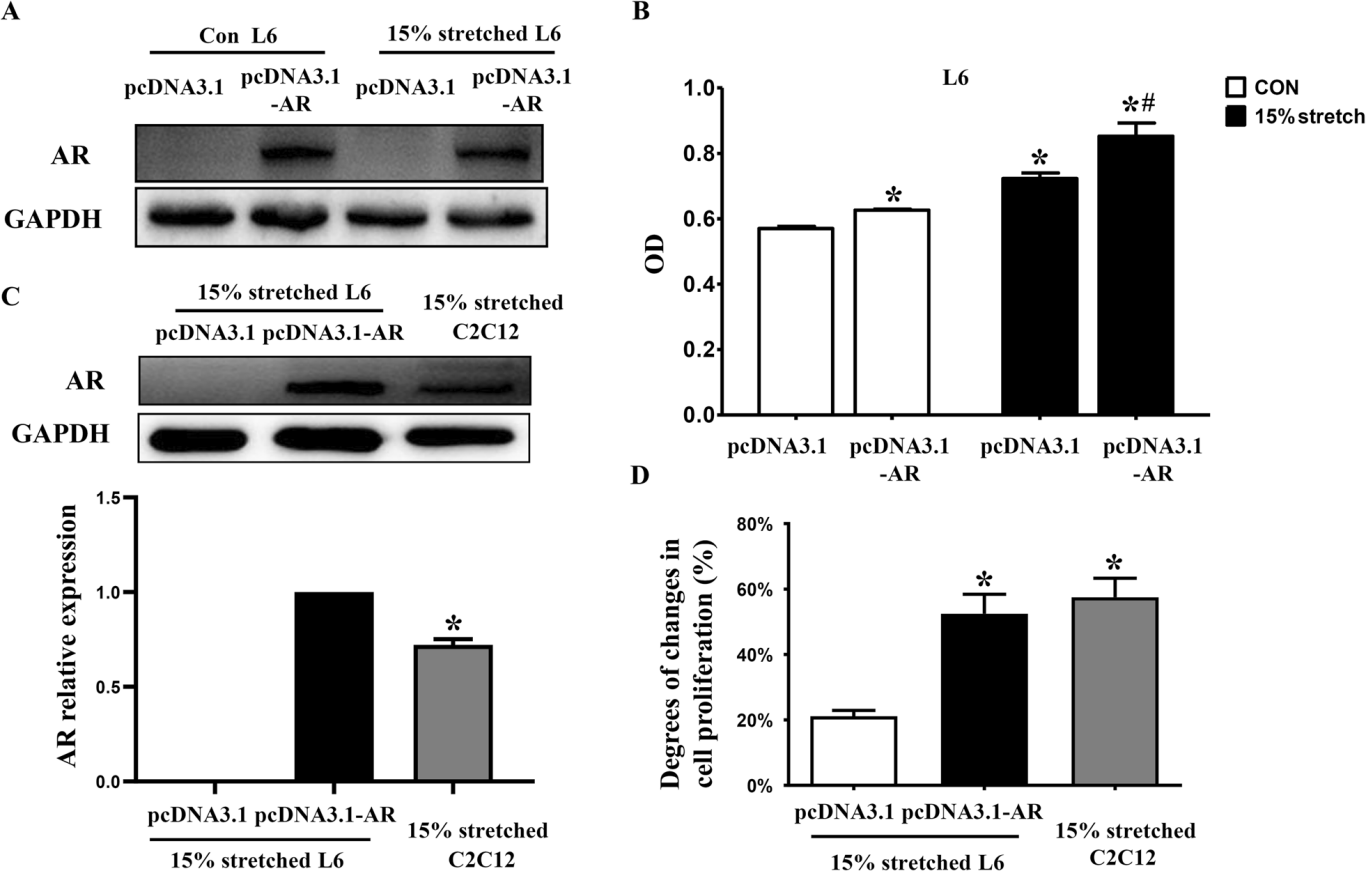
Overexpression of AR further increased 15% stretch-induced pro-proliferation, approaching to the pro-proliferation degree of 15% stretched C2C12 myoblasts. L6 cells were seeded onto flexible-bottomed 6-well plates and transfected with pcDNA3.1 vector or pcDNA3.1-AR recombinant plasmid (2.5 μg/well) using LipoPlus™ reagent. After 24 h incubation, L6 cells were subjected to 15% stretch at 0.5 Hz lasting for 6 h or left un-stretched. After another 24 h incubation, the changes in (**a**) the AR level and (**b**) the proliferation of L6 cells in un-stretched and 15% stretched conditions were compared between transfection with pcDNA3.1-AR recombinant plasmid and with pcDNA3.1 vector. *p < 0.05 vs CON_pcDNA3.1_; ^#^*p* < 0.05 vs 15% stretch_pcDNA3.1_. The differences in (**c**) the AR level and (**d**) the pro-proliferation extents after 15% stretch were compared between L6 myoblasts transfected with pcDNA3.1-AR and C2C12 myoblasts. *p < 0.05 vs 15% stretched L6 myoblasts_pcDNA3.1-AR_ (**c**) or 15% stretched L6 myoblasts_pcDNA3.1_ (**d**). The pro-proliferation extents of 15% stretched L6 and C2C12 cells were calculated using the formula: = (OD_15% stretch_-OD_CON_)/OD_CON_; and for stretched L6 myoblasts transfection with pcDNA3.1-AR recombinant plasmid using the following formula: = (OD_15% stretch+pcDNA3.1-AR_-OD_CON+pcDNA3.1_)/OD_CON+pcDNA3.1_. The experiments were repeated at least three times, and the results were from three independent experiments and represented as mean ± SD

**Fig. 4:**
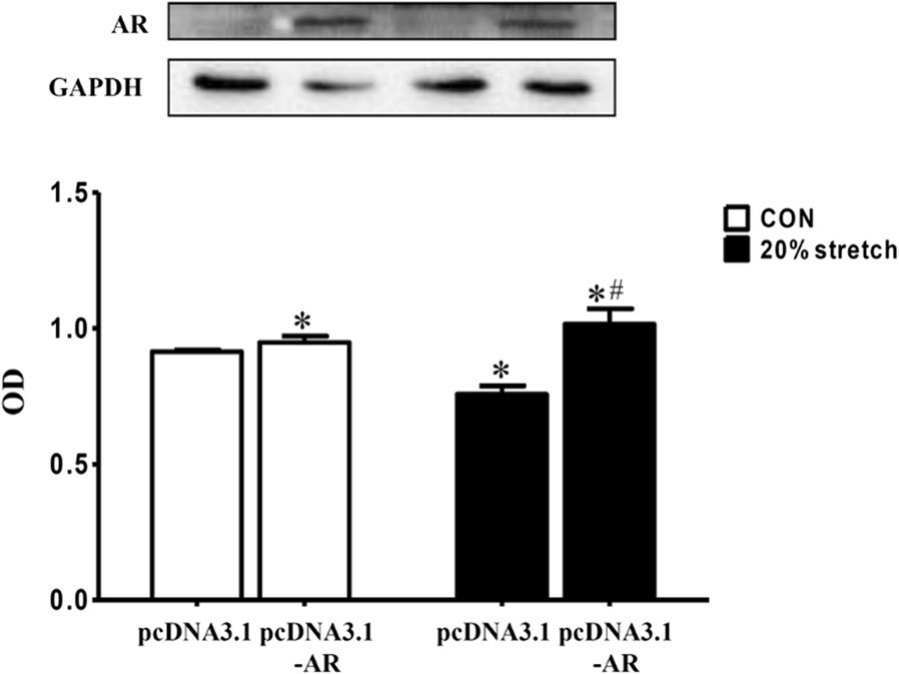
AR overexpression in L6 myoblasts reversed 20% stretch-induced anti-proliferation. L6 myoblasts were seeded onto flexible-bottomed 6-well plates and divided into four groups: CON plus transfected with pcDNA3.1, CON plus transfected with pcDNA3.1-AR, 20% stretch plus transfected with pcDNA3.1, and 20% stretch plus transfected with pcDNA3.1-AR groups. The cells were transfected with pcDNA3.1 vector or pcDNA3.1-AR recombinant plasmid (2.5 μg/well) before exposing to 20% stretch. Then the level of AR protein and cell proliferation were detected at 24 h after stretch finished by Western blot and CCK8, respectively. The results were from three independent experiments and represented as mean ± SD, *p < 0.05 vs CON_pcDNA3.1_; ^#^*p* < 0.05 vs 20% stretch_pcDNA3.1_

To confirm AR’s role in the proliferation of stretched myoblasts, AR overexpression plasmid was transfected into L6 myoblasts before exposing to 15% and 20% stretches. We found AR protein could be obviously detected in L6 myoblasts after transfection with AR overexpression plasmid (Fig. 3a), and AR overexpression further enhanced the proliferation of 15% stretched L6 myoblasts (Fig. 3b) by approximately 50%, reaching similar pro-proliferation degree of 15% stretched C2C12 myoblasts (Fig. 3d) when the level of AR in 15% stretched L6 myoblasts was higher than that of C2C12 myoblasts undertook 15% stretch (Fig. 3c), and reversed the anti-proliferation of 20% stretch on L6 myoblasts (Fig. 4).

### *AR's role in 15% stretch-induced pro-proliferation was fulfilled *via* activating p38 and ERK1/2 rather than PI3K/Akt*

**Fig. 5:**
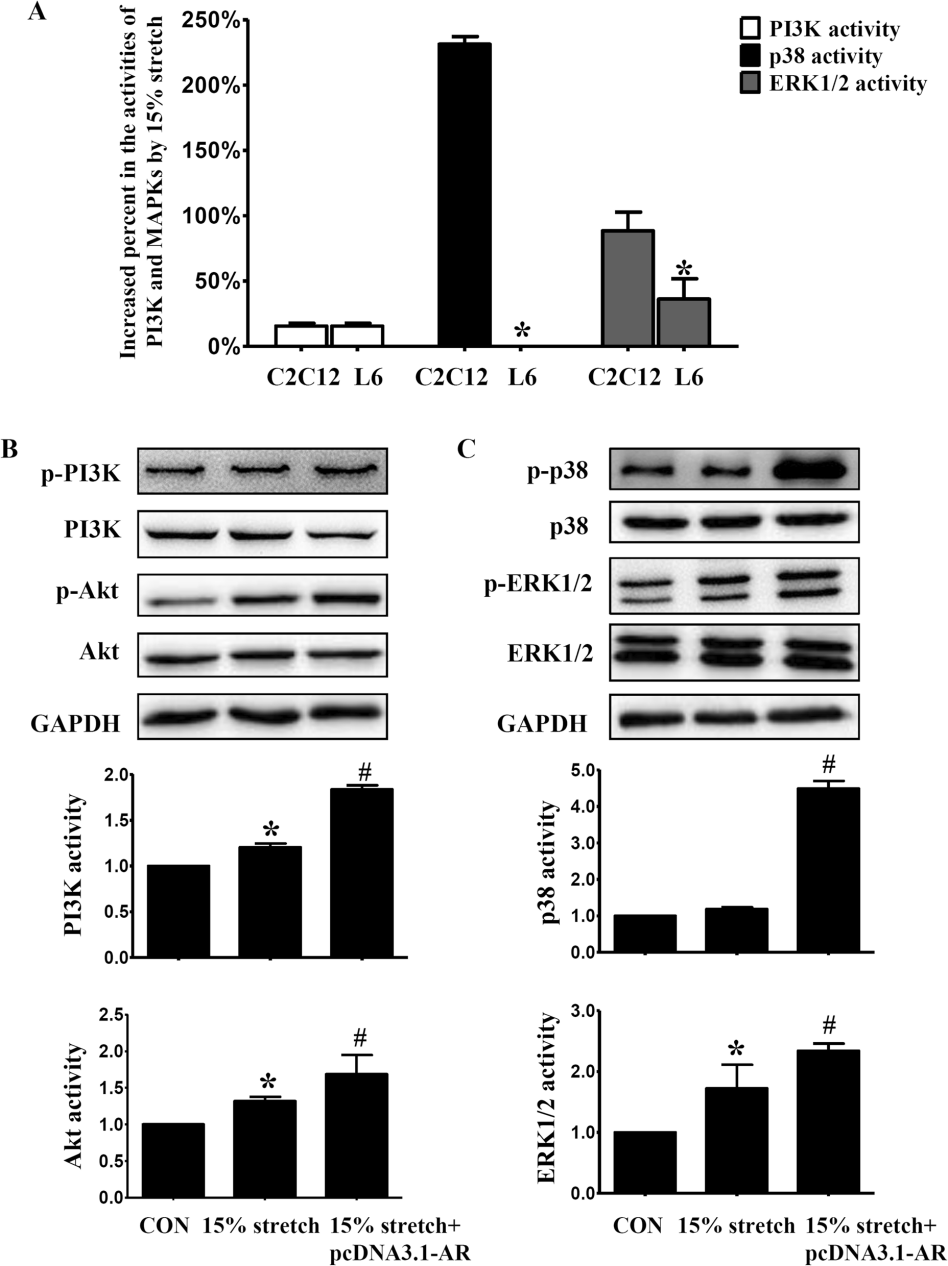
The increased extents of activations in p38, ERK1/2 and PI3K/Akt were different between 15% stretched L6 and C2C12 myoblasts (**a**) and transfection L6 cells with AR overexpression plasmid further enhanced the above molecules' activities (**b** and **c**). **a** Myoblasts C2C12 (with AR) and L6 (no detectable AR) were seeded onto flexible-bottomed 6-well plates and incubated for 24 h prior to 15% stretch. Then cells were collected and the activities of PI3K/Akt, p38 and ERK1/2 (reflected as the ratios of p-PI3K/PI3K, p-Akt/Akt, p-p38/p38 and p-ERK1/2/ERK1/2, respectively) were detected at 24 h after stretch finished. The promoted degrees in the activities of PI3K and MAPKs (p38 and ERK1/2) were calculated by the following formula: = (the activities of PI3K/Akt and MAPKs (p38 and ERK1/2) _15% stretch_—the activities of PI3K/Akt and MAPKs (p38 and ERK1/2) _CON_)/ the activities of PI3K/Akt and MAPKs (p38 and ERK1/2) _CON_. * indicated *p* < 0.05 vs. C2C12 cells. **b** and **c** L6 myoblasts were seeded onto flexible-bottomed 6-well plates one day before transfection, and when the confluence reached ~ 50%, pcDNA3.1 vector or pcDNA3.1-AR overexpression plasmid (2.5 μg/well) was transfected before exposing to 15% stretch, and the activities of PI3K/Akt, p38 and ERK1/2 were detected by Western blot at 24 h after stretch finished. *p < 0.05 vs. CON_pcDNA3.1_; ^#^*p* < 0.05 vs 15% stretch_pcDNA3.1_. The differential values resulting from three independent experiments were compared (mean ± SD, n = 3)

To clarify relevant signal pathways involved in AR's roles in 15% stretch-induced pro-proliferation, the increased degrees of activities of PI3K/Akt, ERK1/2 and p38 were compared between C2C12 and L6 myoblasts subjected to 15% stretch, and we found that there was no difference in PI3K activity between the two cells but the increment of ERK1/2 activity in L6 myoblasts was lower (almost half) than that in C2C12 myoblasts, and what’s more interesting was that p38 activity had no change in 15% stretched L6 cells but about 2 folds increase in 15% stretched C2C12 myoblasts (Fig. 5a). These results indicated that activated ERK1/2 and p38 (especially p38) but not PI3K were associated with AR's pro-proliferative effect on 15% stretched myoblasts.

Furthermore, the activations of those above molecules in L6 cells were detected after AR overexpression plasmid transfection, and we found that accompanied with proliferation increase of L6 cells (Fig. 3), the activities of p38 and ERK1/2, especially p38 activity were remarkably increased (Fig. 5c), demonstrating AR's role in promoting the proliferation of 15% stretched myoblasts was via activating p38 and ERK1/2. In addition, although there was no difference in 15% stretch-induced increase in PI3K/Akt activations between C2C12 and L6 myoblasts, exogenous AR could further increase the activities of PI3K/Akt in 15% stretched L6 myoblasts (Fig. 5b).

### Effect of decreased AR on 20% stretch-induced anti-proliferation was mediated by inhibiting p38 instead of ERK1/2 and PI3K/Akt

**Fig. 6:**
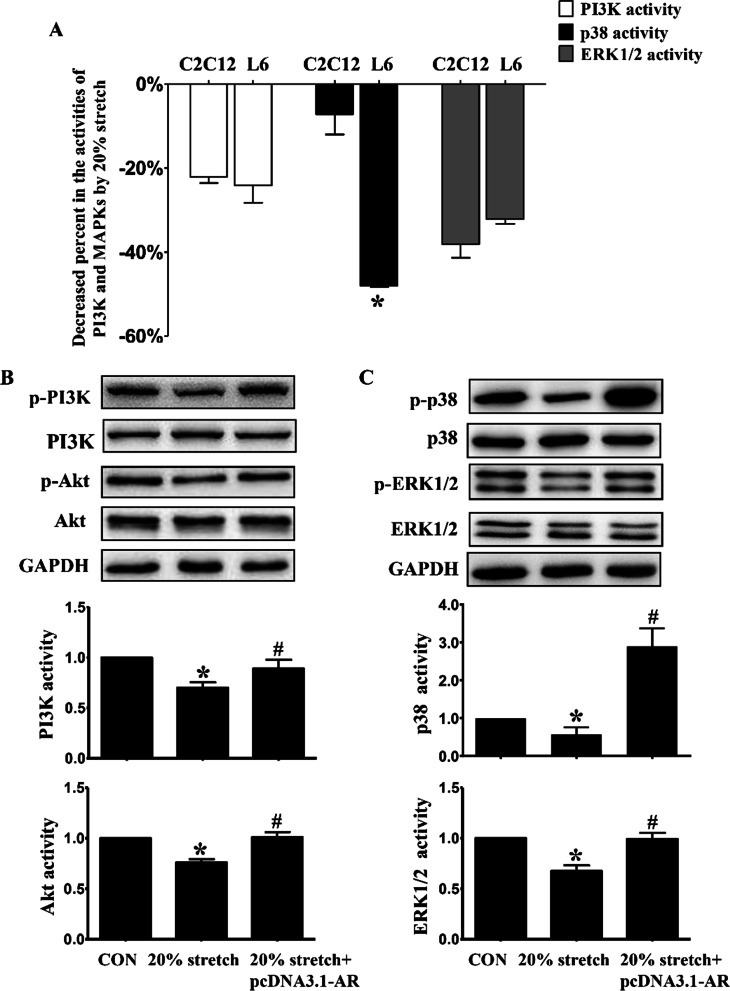
The decreased extents of activations in p38, ERK1/2 and PI3K/Akt were different between 20% stretched L6 and C2C12 myoblasts **a** and transfection L6 cells with AR overexpression plasmid reversed the above molecules' activities (**b** and **c**). **a** C2C12 and L6 cells were seeded onto flexible-bottomed 6-well plates and incubated for 24 h prior to 20% stretch. Then cells were collected and the activities of PI3K/Akt, p38 and ERK1/2 (reflected as the ratios of p-PI3K/PI3K, p-Akt/Akt, p-p38/p38 and p-ERK1/2/ERK1/2, respectively) were detected by Western blot at 24 h after stretch finished. The decreased extents in activities of PI3K, p38 and ERK1/2 were calculated using the following formula: = (the activities of PI3K/Akt and MAPKs (p38 and ERK1/2) _20% stretch_—the activities of PI3K/Akt and MAPKs (p38 and ERK1/2) _CON_)/ the activities of PI3K/Akt and MAPKs (p38 and ERK1/2) _CON_. * indicated *p* < 0.05 vs. C2C12 cells. **b** and **c** L6 myoblasts were seeded onto flexible-bottomed 6-well plates, when the confluence reached ~ 50%, pcDNA3.1 vector or pcDNA3.1-AR overexpression plasmid was transfected into L6 myoblasts before subjected to 20% stretch, and the activities of PI3K/Akt, p38 and ERK1/2 were detected at 24 h after stretch. *p < 0.05 vs. CON_pcDNA3.1_; ^#^*p* < 0.05 vs 20% stretch_pcDNA3.1_

To identify relevant signal molecules involving in decreased AR's roles in 20% stretch-induced anti-proliferation on myoblasts, we compared the discrepancy between C2C12 and L6 myoblasts subjected to 20% stretch in the activities of PI3K, p38 and ERK1/2, and found that the attenuated degree of p38 activity by 20% stretch was higher in L6 myoblasts than that in C2C12 myoblasts (approximately 5.7 folds), while the decreases of PI3K and ERK's activities resulted from 20% stretch were similar to that in C2C12 cells (Fig. 6a), which indicated that the activation of p38 but not PI3K and ERK was related to decreased AR's anti-proliferation on 20% stretched myoblasts.

Furthermore, transfection with AR overexpression plasmid into 20% stretched L6 myoblasts led to an enormous enhancement in p38 activity while mild increases in the activations of PI3K, Akt and ERK1/2 (Fig. 6b and C), demonstrated that the anti-proliferative effect of decreased AR on 20% stretched myoblasts was via inhibiting p38 activation (instead of ERK1/2 and PI3K's activations).

### AR overexpression up-regulated IGF-1R levels but not IGF-1 secretion of L6 myoblasts subjected to 15% or 20% stretch

**Fig. 7:**
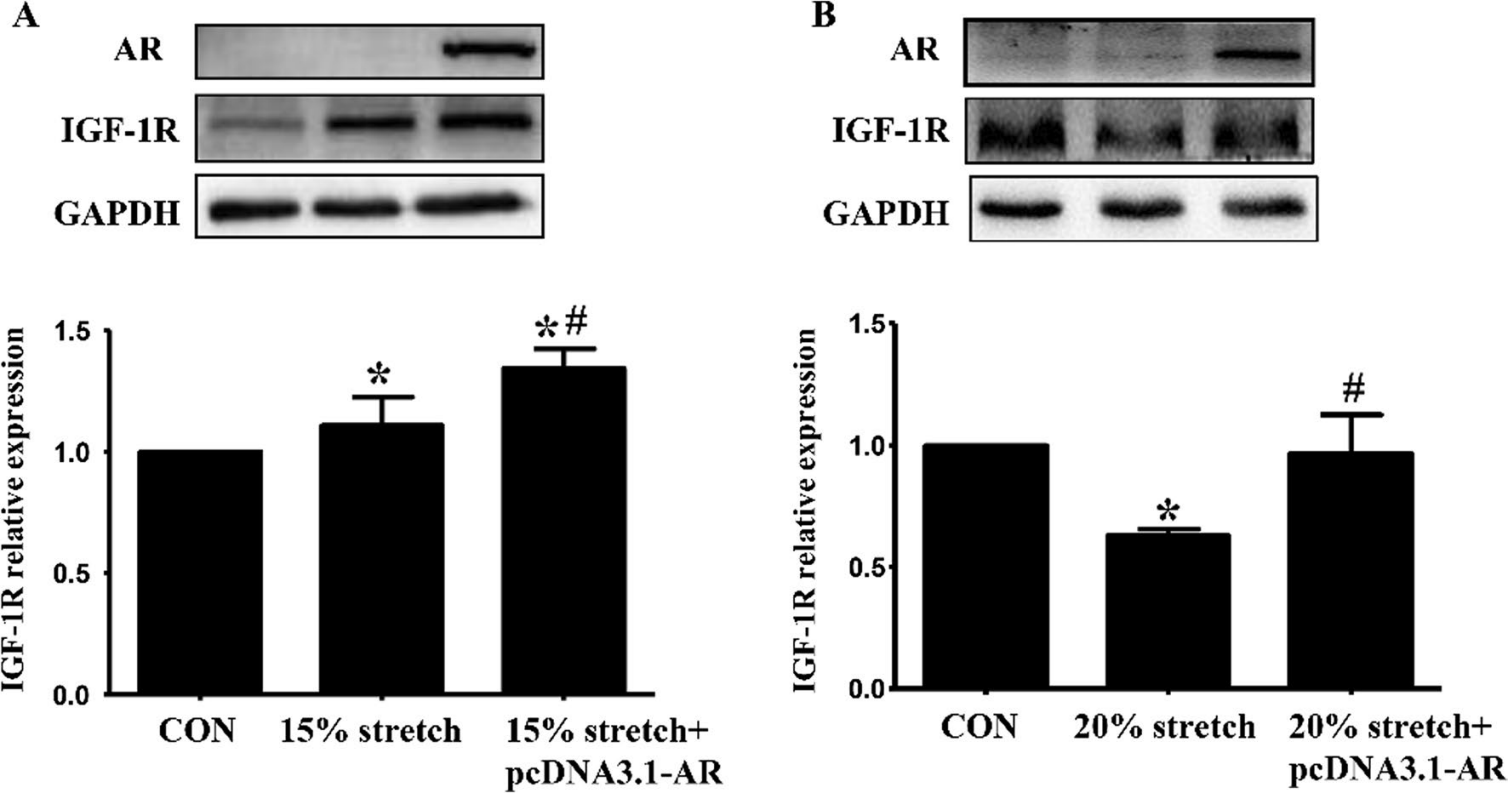
AR overexpression up-regulated the levels of IGF-1R in L6 myoblasts undertook 15% or 20% stretch. L6 myoblasts were seeded onto flexible-bottomed 6-well plates and transfected with AR overexpression plasmid before subjected to 15% or 20% stretch, then the protein levels of IGF-1R were determined at 24 h after stretch finished. The results resulting from three independent experiments were analyzed and represented as mean ± SD. *p < 0.05 vs. CON and ^#^*p* < 0.05 vs 15% stretch or 20% stretch

IGF-1 secretion from L6 myoblasts undertook 15% or 20% stretch were determined after transfection with AR overexpression plasmid, and still no detectable IGF-1 was secreted. For IGF-1R, significant increase and decrease of IGF-1R protein levels were observed in L6 myoblasts subjected to 15% (Fig. 7a) and 20% (Fig. 7b) stretches, respectively. AR overexpression enhanced the IGF-1R levels in both 15% and 20% stretched L6 myoblasts (Fig. 7).

### Exogenous IGF-1 recombinant polypeptide further increased IGF-1R protein level and the activities of PI3K/Akt and MAPKs (p38 and ERK1/2) in 15% stretched L6 myoblasts, accompanied with the enhanced proliferation

**Fig. 8:**
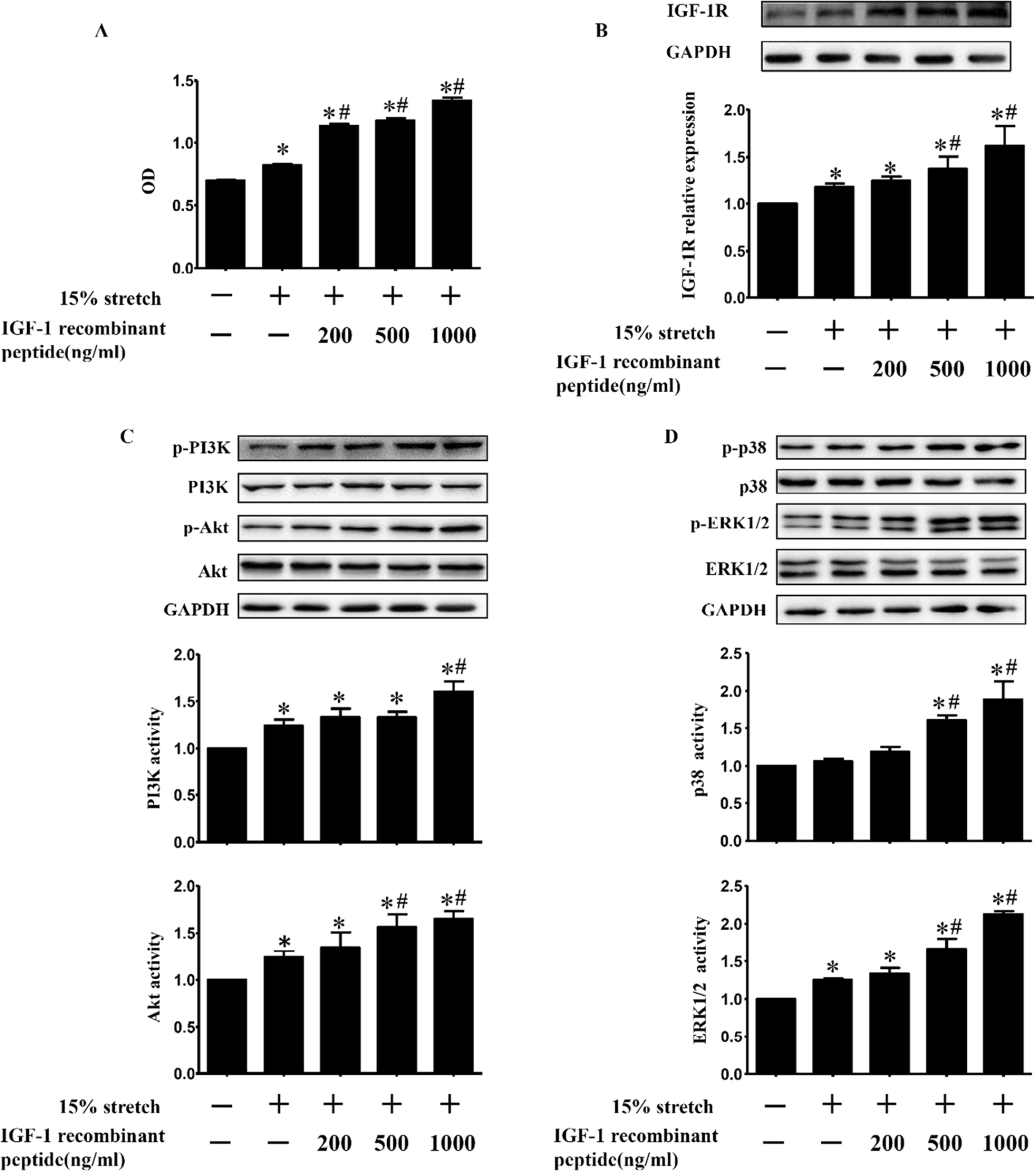
Exogenous IGF-1 recombinant polypeptide further promoted the proliferation of 15% stretched L6 myoblasts, accompanied with the increases in the protein level of IGF-1R as well as the activities of PI3K/Akt and MAPKs (p38 and ERK1/2) in a dose-dependent manner. L6 myoblasts were divided into five groups (CON, 15% stretch, and 15% stretch administrated with 200, 500 and 1000 ng/ml IGF-1 recombinant polypeptide), and IGF-1 recombinant polypeptide at different concentrations were added into L6 cell culture medium at 1 h prior to 15% stretch, and cell proliferation were detected by CCK8 (A); the protein level of IGF-1R (B), and the activities of PI3K/Akt (C) and MAPKs (D) were detected by Western blot at 24 h after stretch finished. The results were from three independent experiments, and the data were analyzed and represented as mean ± SD. *p < 0.05 vs. CON; ^#^*p* < 0.05 vs 15% stretch

Exogenous IGF-1 recombinant polypeptide with various concentrations (200, 500, and 1000 ng/ml) were incubated with cells to verify the regulation of IGF-1 on the proliferation as well as PI3K/Akt, p38 and ERK1/2 signals in 15% stretch myoblasts. As shown in Fig. 8, IGF-1 recombinant polypeptide further promoted the proliferation of 15% stretched L6 myoblasts in a dose-dependent manner (Fig. 8a), accompanied with dose-dependent increases in protein level of IGF-1R (Fig. 8b) and in activities of PI3K, Akt, p38 and ERK1/2 (increased phosphorylated protein levels of PI3K, Akt, p38 and ERK1/2) (Fig. 8c and d).

## Discussion

### AR's roles in the proliferation of myoblasts undertook appropriate stretch and excessive stretch

Satellite cells or myoblasts exert crucial roles in muscle hypertrophy, and impairments in the proliferation and differentiation of these cells lead to muscle atrophy. Exercise-induced muscle hypertrophy is also associated with the increased proliferation of satellite cells [27], furthermore, satellite cell depletion blocked exercise-induced muscle hypertrophy[28, 29]. In vitro, exposing satellite cells or myoblasts to mechanical stretch is usually used to mimic the stimulation of exercise on muscle, and multiple lines of evidences in vitro including our previous work have demonstrated that appropriate mechanical stretch with several deformations (10%, 15% or 17%), frequencies (0.25 Hz or 0.5 Hz) and durations (1 h or 2 h) promoted the proliferation of primary satellite cells or mouse C2C12 myoblasts[17, 30], while excessive mechanical stretch inhibited the proliferation of the above cells when deformation increased up to 20%[17], even led to cell apoptosis[31], so 15% and 20% stretches are chosen in our experiments to mimic appropriate exercise and overtraining respectively.

As mentioned in the Introduction, our previous work revealed that in C2C12 myoblasts (expressed AR), 15% stretch-induced pro-proliferation was likely to be mediated by up-regulation of AR because AR specific antagonist flutamide attenuated the pro-proliferation of 15% stretch on C2C12 myoblasts in a dose-dependent manner. Meanwhile, 20% stretch-induced anti-proliferation was accompanied with the down-regulation of AR [17]. What's interesting is that another commonly used L6 myoblasts, which has no detectable AR, reported to promoted and inhibited proliferation after 15% and 20% stretches (at 0.5 Hz lasting for 6 h) respectively [26], showed different proliferative characteristics-lower pro-proliferation by 15% stretch and higher anti-proliferation by 20% stretch compared with C2C12 myoblasts, which has AR expression. Furthermore, transfection with AR overexpression plasmid significantly enhanced the pro-proliferation of 15% stretch on L6 cells (closing to the identical level of 15% stretched C2C12 cells, at that time the level of AR in 15% stretched L6 cells was higher than that of 15% stretched C2C12 myoblasts), and totally reversed the anti-proliferation of 20% stretch on L6 cells. These results demonstrated key and indispensable effects of elevated AR in 15% stretch-induced pro-proliferation of myoblasts and of reduced AR in 20% stretch-induced anti-proliferation of myoblasts, despite it’s not the unique factor for stretch-regulated myoblast proliferation.

In addition, under un-stretched condition, the proliferation rate of L6 myoblasts was lower than that of C2C12 myoblasts (about 80.6% and 72.5% of C2C12 myoblasts at 24 h and 48 h after seeding). And AR knockdown by siRNA inhibited the proliferation of C2C12 myoblasts, while transfection with AR overexpression plasmid increased the proliferation of L6 myoblasts, implied the important role of AR in proliferation of un-stretched myoblasts.

### The mechanisms of AR's effects on appropriate and excessive stretches-regulated myoblast proliferation

It is well known that AR affects muscle mass through genomic and non-genomic mechanisms. Recently, non-genomic mechanism of AR has been proven to be crucial in promoting myoblast proliferation and increasing muscle mass through interactions with other signaling molecules such as IGF-1 and its downstream molecules[32]. PI3K/Akt and MAPKs (p38 and ERK1/2) are the common downstream molecules of IGF-1, and IGF-1 promotes the proliferation of primary satellite cells or myoblast via activating PI3K/Akt and MAPKs (p38 and ERK1/2), specific inhibitors of PI3K (LY294002), p38 (SB203580) and ERK1/2 (U0126) blocked the pro-proliferative effect of IGF-1[17, 19, 21]. In addition, several miRNA-induced myotube atrophy and proliferation inhibition of bovine myoblasts were related to down-regulated IGF-1, thus suppressing PI3K/Akt signal pathway[33, 34].

Not only that, recent studies showed the importance of PI3K/Akt, p38 and ERK1/2 MAPKs in the anabolic action of AR, which was partly fulfilled through the crosstalk with AR[35–37].Testosterone promotes muscle hypertrophy via activating PI3K/Akt[35], ERK1/2[36] and p38 MAPK[37], and administration of specific inhibitors of PI3K (LY294002) and p38 (SB203580) block the testosterone's effects. In fact, in addition to AR-induced myotube hypertrophy, testosterone-induced differentiation (increases of myotube number and diameter) of fusion impaired C2C12 myoblasts is also associated with the activation of PI3K/Akt[38] and androgen/AR-participated skeletal muscle glucose metabolism is related to the activations of Akt and ERK1/2 signal pathways [39].

The above situations are involved in non-exercise conditions or un-stretched myoblasts, what about the mechanisms of AR in appropriate stretch and overstretch condition? Whether IGF-1-mediated PI3K/Akt and MAPKs (p38 and ERK1/2) pathways still play key roles in the effects of AR on the two stretches-modulated proliferation of myoblasts and does there exist difference with respect to signal pathways involving in 15% and 20% stretches-regulated proliferation of myoblasts? Our previous work in 15% stretched C2C12 myoblasts indicated that the effect of increased AR on pro-proliferation was fulfilled by IGF-1 mediated activations of PI3K/Akt, p38 and ERK1/2 using IGF-1 neutralized antibody and specific inhibitors of PI3K/Akt, p38 and ERK1/2[17]; for 20% stretch-induced anti-proliferation of C2C12 myoblasts, the decrease of AR might exert its role through inhibiting IGF-1 secretion using IGF-1 recombinant polypeptide[17]. However, the AR's effects on 15% and 20% stretches-modulated proliferation of myoblasts and the mechanisms require further confirmation.

So in this paper the discrepancies between myoblasts L6 (without AR) and C2C12 (with AR) were compared in the proliferation, the levels of IGF-1/IGF-1R, and the levels and activations of PI3K/Akt, p38 and ERK1/2 after 15% or 20% stretch, then the above indicators in L6 myoblasts were detected again after transfection with AR overexpression plasmid or treatment with IGF-1 recombinant polypeptide. Except for similar elevated degree of PI3K activity induced by 15% stretch between L6 and C2C12 myoblasts, obvious differences were found between the two myoblasts after 15% stretch, including: (1) IGF-1: increased secretion in C2C12 myoblasts while no secretion in L6 myoblasts; (2) ERK1/2 activity: increased degree in L6 myoblasts was half of that in C2C12 myoblasts; (3) p38 activity: no change in L6 myoblasts but beyond threefold elevation in C2C12 myoblasts. Furthermore, transfection L6 myoblasts with AR overexpression plasmid and treatment with IGF-1 recombinant polypeptide both enhanced the level of IGF-1R (although still no IGF-1 secretion), and the activations of p38 and ERK1/2 in 15% stretched L6 myoblasts, accompanied with the further promotion of L6 cell proliferation. These results indicated that the pro-proliferative effect of AR on 15% stretched myoblasts was mediated through increasing IGF-1R, thus activating p38 and ERK1/2, especially p38, rather than PI3K/Akt pathway.

For 20% stretch, our previous studies revealed significant decreases including the secretion of IGF-1 and the activations of PI3K/Akt, p38 and ERK1/2 in 20% stretched C2C12 myoblasts[17]. In the present study, there was no difference in the inhibition degree of PI3K/Akt and ERK1/2's activations between 20% stretched L6 and C2C12 myoblasts, but remarkable discrepancies were found after 20% stretch between the two myoblasts, including: (1) IGF-1: decreased secretion in C2C12 myoblasts while still no secretion in L6 myoblasts; (2) p38 activity: about 6.7-fold decrease in L6 myoblasts than C2C12 myoblasts. Furthermore, transfection with AR overexpression plasmid and IGF-1 recombinant polypeptide both increased the level of IGF-1R and the activity of p38, accompanied with the reverse of 20% stretch-induced anti-proliferation of L6 myoblasts. The above results indicated that declined AR mediated the proliferation inhibition of 20% stretch on myoblasts, which was achieved via suppressing IGF-1R, thus inhibiting p38 activation.

In combination with our another work which demonstrated the important roles of PI3K/Akt and MAPK (ERK1/2 and p38) in stretches-modulated proliferation of L6 myoblasts [26], the present study indicated that the pro-proliferation of 15% stretch through activating AR- IGF-1/IGF-1R- p38 and ERK1/2 pathways while anti-proliferation of 20% stretch through inhibiting AR- IGF-1/IGF-1R -p38 pathway. It is interesting to find a discrepancy in signal pathways fulfilling AR's role in 20% and 15% stretches modulated myoblast proliferation (inhibiting AR- IGF-1R- p38 pathway vs activating AR- IGF-1R- p38 and ERK1/2 pathway). This study is beneficial to understand in depth the role and mechanisms of AR on appropriate exercise increases while excessive exercise decreases muscle mass.

## Conclusions

The present study demonstrated AR's crucial roles in stretches regulated proliferation of myoblasts, and increased AR fulfilled appropriate stretch's pro-proliferation via activating IGF-1R-p38 and ERK1/2 pathways while decreased AR achieved excessive stretch's anti-proliferation via inhibiting IGF-1R- p38 pathway.

## Data Availability

The datasets used and/or analyzed during the current study are available from the corresponding author on reasonable request.

## Abbreviations

AR: Androgen receptor
AREs: Androgen response elements
IGF-1: Insulin-like growth factor
IGF-1R: IGF-1 receptor
PI3K: Phosphoinositide 3-kinases
Akt: Protein kinase B
MAPK: Mitogen-activated protein kinase
ERK1/2: Extracellular signal-regulated kinases 1 and 2
